# Zn-Driven Discovery of a Hydrothermal Vent Fungal Metabolite Clavatustide C, and an Experimental Study of the Anti-Cancer Mechanism of Clavatustide B

**DOI:** 10.3390/md12063203

**Published:** 2014-05-28

**Authors:** Panpan Ye, Ling Shen, Wei Jiang, Ying Ye, Chen-Tung Arthur Chen, Xiaodan Wu, Kuiwu Wang, Bin Wu

**Affiliations:** 1 Eye Center, The Second Affiliated Hospital, Zhejiang University School of Medicine, Hangzhou 310000, China; E-Mails: yepanpan@hotmail.com (P.Y.); zzynwk@126.com (L.S.); 2 Ocean College, Zhejiang University, Hangzhou 310058, China; E-Mails: jw6912@163.com (W.J.); gsyeying@zju.edu.cn (Y.Y.); ctchen@faculty.nsysu.edu.tw (C.-T.A.C.); wxd_zju@163.com (X.W.); 3 Institute of Marine Geology and Chemistry, National Sun Yat-sen University, Kaohsiung 80424, Taiwan; 4 Department of Applied Chemistry, Zhejiang Gongshang University, Hangzhou 310058, China; E-Mail: wkwnpc@zjgsu.edu.cn

**Keywords:** clavatustides, stress-driven discovery, hydrothermal vent, anti-cancer

## Abstract

A naturally new cyclopeptide, clavatustide C, was produced as a stress metabolite in response to abiotic stress elicitation by one of the hydrothermal vent fluid components Zn in the cultured mycelia of *Aspergillus clavatus* C2WU, which were isolated from *Xenograpsus testudinatus*. *X. testudinatus* lives at extreme, toxic habitat around the sulphur-rich hydrothermal vents in Taiwan Kueishantao. The known compound clavatustide B was also isolated and purified. This is the first example of a new hydrothermal vent microbial secondary metabolite produced in response to abiotic Zn treatment. The structures were established by spectroscopic means. The regulation of G1-S transition in hepatocellular carcinoma cell lines by clavatustide B was observed in our previous study. The purpose of the present study was to verify these results in other types of cancer cell lines and elucidate the possible molecular mechanism for the anti-cancer activities of clavatustide B. In different human cancer cell lines, including pancreatic cancer (Panc-1), gastric cancer (MGC-803), colorectal cancer (SW-480), retinoblastoma (WERI-Rb-1) and prostate cancer (PC3), clavatustide B efficiently suppressed cell proliferations in a dose-dependent manner. Although different cancer cell lines presented variety in Max effect dose and IC_50_ dose, all cancer cell lines showed a lower Max effect dose and IC_50_ dose compared with human fibroblasts (hFB) (*p* < 0.05). Moreover, significant accumulations in G1 phases and a reduction in S phases (*p* < 0.05) were observed under clavatustide B treatment. The expression levels of 2622 genes including 39 cell cycle-associated genes in HepG2 cells were significantly altered by the treatment with 15 μg/mL clavatustide B after 48 h. *CCNE2* (cyclin E2) was proved to be the key regulator of clavatustide B-induced G1-S transition blocking in several cancer cell lines by using real-time PCR.

## 1. Introduction

Ocean hydrothermal vent microorganisms adapt and respond rapidly to changes in the concentrations and availability of metals within their environment [1]. In the previous study, we discovered two structurally interesting hepatocellular carcinoma cell inhibitory cyclodepsipeptides from a hydrothermal vent fungus [2], which we isolated from extreme, toxic habitat around the metal-rich hydrothermal vents in Taiwan Kueishantao [3]. The chemical diversity of the secondary metabolites from marine fungi is considerably high [4,5]. New strategies to discover the novel bioactive compounds including biotic [6,7,8] and abiotic [4,9] stress elicitations have been applied. In this study, one naturally new cyclopeptide was produced as a stress metabolite in the cultured mycelia of hydrothermal fungus *Aspergillus clavatus* C2WU in response to abiotic Zn stress elicitation. Zn is one of the hydrothermal vent fluid components [10].

Cancer is a major public health problem and the leading cause of death worldwide. According to the data from the National Cancer Institute of United States [11], more than 1.6 million new cancer cases and a half million cancer deaths had been projected to occur in the United States in 2014 [11]. In China, there are 3.5 million newly diagnosed cancer patients and 2.5 million cancer deaths every year according to a recently released annual report from the National Cancer Prevention and Control Office. Comprehensive cancer therapy, including surgery, chemo- and radio-therapy and traditional Chinese Medicine has been advocated in recent years [12,13] in order to cure cancer or considerably prolong life while improving the quality of life. Some cancer types, including lung cancer, colorectal cancer, breast cancer, and prostate cancer have higher cure rates and declined death rates when detected early and treated with best practices. However, some other common cancer types, such as hepatobiliary and pancreatic cancer have higher death rates at similar detection time and treatment [11]. Therefore, the discovery of novel and potent anti-cancer drugs is desperately needed. We previously found that clavatustide B regulated G1-S transition in liver cancer cell lines [2]. In this study we presented the results obtained from other types of cancer cell lines. This study was also performed to elucidate the possible molecular mechanism of the anti-cancer activities of clavatustide B.

## 2. Results and Discussion

### 2.1. Structural Elucidation of the Stress Metabolite

**Chart 1:**
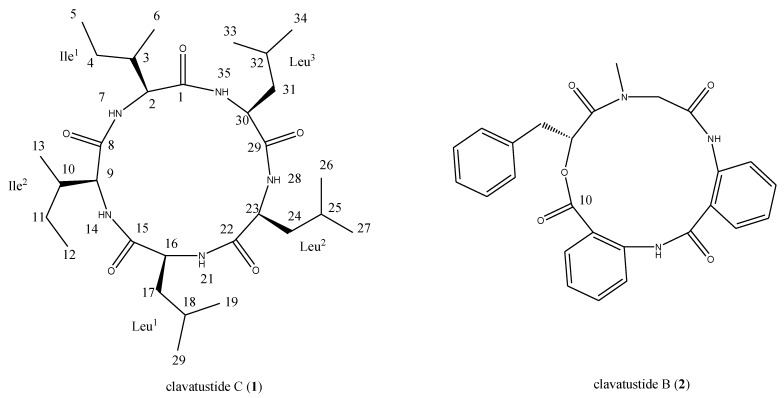
Structures of clavatustides B and C.

The hydrothermal fungus *A. clavatus* C2WU isolated from *X. testudinatus*, was cultured in the absence and presence of the abiotic stress agent, ZnSO_4_. Upon comparison of TLC plates of mycelia extracts from both conditions, an additional spot in the extract of ZnSO_4_ treated culture was detected. The compound was putatively produced in response to abiotic stress. The stress metabolite clavatustide C was isolated by preparative TLC, and purified by Sephadex LH-20 column chromatography (Chart 1). Clavatustide B produced both in the normal and Zn treated culture condition was isolated using preparative HPLC (Chart 1). 

**Figure 1:**
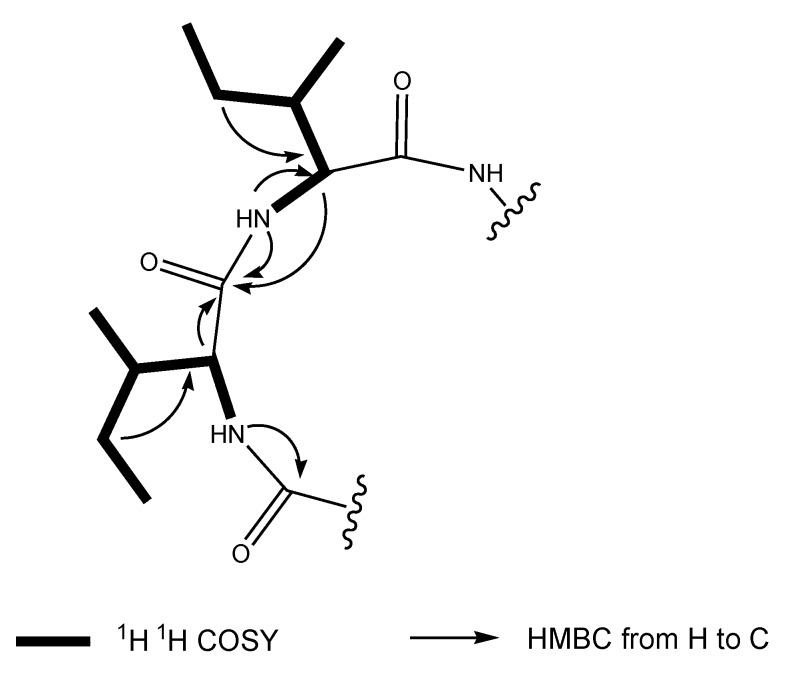
Key 2D NMR correlations of **1**.

Clavatustide C (**1**) was obtained as white powder. The HR-TOF-MS exhibited an ion peak at *m*/*z* 566.4279 [M + H]^+^ (calcd. 566.4276), corresponding to the molecular formula, C_30_H_55_N_5_O_5_. The ^1^H and ^13^C NMR spectra for **1** exhibited resonances typical of a cyclic peptide (Table 1). In the ^13^C NMR spectrum recorded in DMSO-*d*_6_, five carbonyl resonances at δ_C_ 170.2, 172.6, 171.2, 171.2 and 172.0, respectively, were observed, suggestive of structural feature of pentapeptide. The peptide nature of the molecule was further supported by the presence of five NH protons in the ^1^H NMR spectrum (δ_H_ 8.22, 8.35, 8.63, 7.87, 7.30, respectively) and five α-CH signals (δ_C_ 58.3, δ_H_ 4.04; δ_C_ 62.9, δ_H_ 3.34; δ_C_ 52.1, δ_H_ 4.31; δ_C_ 51.5, δ_H_ 4.31; δ_C_ 50.3, δ_H_ 4.41, respectively). Analysis of the 1D and 2D NMR data allowed the assignment of two isoleucine and three leucine residues in the molecule of **1**. The most common cyclic sequence of these five peptide units is cyclo-(l-Leu-l-Ileu-l-Leu-l-Ile-l-Lue), viscumamide [14,15]. Two isoleucines are embedded among three leucines in the structure of viscumamide. However, the α-CH carbon resonance was observed to upshift about 4 ppm when compared the NMR data of **1** with those of viscumamide reported [14,15]. The diagnostic HMBC correction from the NH proton signal of one of the isoleucines at δ_H_ 8.22 (NH-7, d, *J* = 8.7 Hz) to the carbonyl carbon signal of the remaining isoleucine at δ_C_ 172.6 (C-8, s) linked two isoleucines by a peptide bond. Key long range correlations were shown in the Figure 1. Thus, the stress metabolite was elucidated as cyclo-(l-Ileu-l-Ile-l-Leu-l-Lue-l-Leu). The absolute configuration of l-isoleucine and l-leucine was determined by the analysis of acidic hydrolysates through a chiral HPLC column, when compared with the authentic l-isoleucine and l-leucine. Although this compound was synthesized by Sakurai and coworkers [15], this is the first time that the cyclic pentapeptide is isolated from a natural source. Since no trivial name has been given, it was, therefore, named as clavatustide C. This is also the first example of Zn-induced cyclic pentapeptide from marine fungi. Since the NMR data of compound **2** were in good agreement with previously reported, compound **2** was identified as the known compound clavatustide B [2].

**Table 1:** NMR Data (500 MHz) for clavatustide C in DMSO-*d*_6_.

Position	δ_C_^a,b^, multiplicities	δ_H_^c^, multiplicities (*J* in Hz)	Position	δ_C_^a,b^, multiplicities	δ_H_^c^, multiplicities (*J* in Hz)
Ile ^1^			Leu^2^		
1	170.2, C		22	171.2, C	
2	58.3, CH	4.04, dd (8.2, 3.0)	23	51.5, CH	4.31, m
3	35.5, CH	1.88, m	24	41.5, CH_2_	1.46, m
4	24.2, CH_2_		25	24.3, CH ^d^	1.48, overlap
5	11.3, CH_3_	0,82, overlap	26	22.8, CH_3_^e^	0.87, overlap
6	15.6, CH_3_	0.83, overlap	27	22.2, CH_3_	0.87, overlap
7	NH	8.22, d (8.7)	28	NH	7.30, d (8.3)
Ile ^2^			Leu^3^		
8	172.6, C		29	172.0, C	
9	62.9, CH	3.34, m	30	50.3, CH	4.41, m
10	33.2, CH	2.29, m	31	38.8, CH_2_	1.46, m
11	25.1, CH_2_	1.48, overlap	32	24.6, CH ^d^	1.48, overlap
12	9.8, CH_3_	0.79, t (7.5)	33	22.4, CH_3_^e^	0.87, overlap
13	15.4, CH_3_	0.83, overlap	34	22.2, CH_3_	0.87, overlap
14	NH	8.35, d (8.4)	35	NH	7.87, d (8.4)
Leu ^1^					
15	171.2, C				
16	52.1, CH	4.31, m			
17	40.1, CH_2_	1.51, m			
18	24.6, CH ^d^	1.49, overlap			
19	22.4, CH_3_^e^	0.87, overlap			
20	22.2, CH_3_	0.87, overlap			
21	NH	8.63, d (8.2)			

^a^ Recorded at 125 MHz; ^b^ Multiplicities inferred from DEPT and HSQC experiments; ^c^ Recorded at 500 MHz; ^d,e^ Interchangeable.

### 2.2. Clavatustide B Inhibits Growth of Cancer Cells

**Figure 2:**
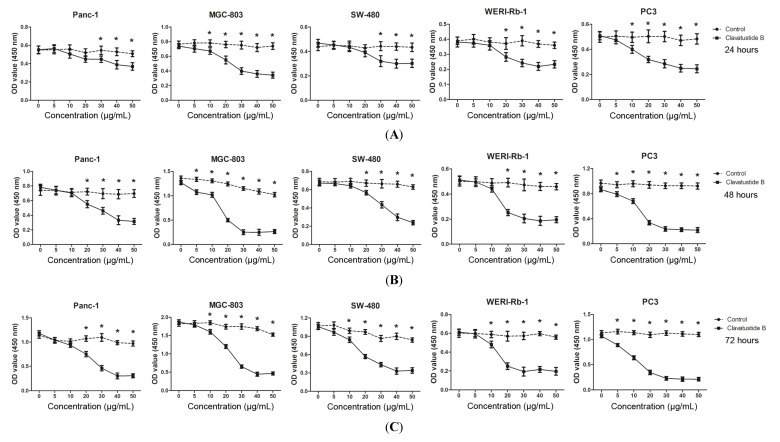
Clavatustide B inhibits growth of different cancer cells in a dose-dependent manner after 24 h (**A**); 48 h (**B**) and 72 h (**C**) treatments. * *p* < 0.05.

Our previous study showed that clavatustide B possessed a preliminary anti-proliferative effect on three hepatocellular cacinoma cell lines (HepG2, SMMC-7721 and Bel-7402) in a dose- and time-dependent manner [2]. This finding prompted us to further investigate the role of clavatustide B in other cancer cell lines, including human pancreatic cancer (Panc-1), gastric cancer (MGC-803), colorectal cancer (SW-480), retinoblastoma (WERI-Rb-1) and prostate cancer (PC3). The results showed that the clavatustide B treatment group exhibited an anti-proliferative effect in a dose-dependent manner in all five cell lines compared with the blank control (Figure 2). Viable cell number reduction became significant at 10–20 μg/mL and the Max effect dose was 40–50 μg/mL in the cancer cell lines after 24 h treatment. From 24 h to 72 h, viable cell number increased at low dose drug treatment (0–10 μg/mL) by comparison of OD values. In contrast, viable cell number at 40–50 µg/mL remained essentially identical between 24 h, 48 h and 72 h for each cell line. The OD values of MGC-803 cells increased from 0.7, 1.3 to 1.8 at zero drug treatment for 24 h, 48 h and 72 h, indicating that the cells continued to proliferate during the 72 h. However, the OD values at 40–50 μg/mL treatment during the same period remained fairly constant at 0.3, suggestive of an inhibition of cell proliferation. The predicted 50% inhibitory concentrations (IC_50_) doses, which are determined as the midpoint between the zero cell killing and the maximal inhibitory effects, were all about essentially identical at 15–20 μg/mL for MGC-803 cell line at each time point. Similar observations were observed with the other four cell lines. The cell viabilities were reduced by 65%, 74%, 63%, 67% and 78% in Panc-1, MGC-803, SW-480, WERI-Rb-1 and PC3 cells, respectively, at a concentration of 40 μg/mL clavatustide B after 72 h treatments.

The results demonstrated that clavatustide B induced complete cessation of cell growth in various types of cancer cells in a short time at 40–50 µg/mL dose levels. The biological effect was dose-dependent but might not time-dependent. It should be noted that Panc-1 is one of the most frequently studied pancreatic cancer cell lines and is chemo- and radio-therapy resistant [16]. The prostate cancer cell line PC3 was also reported to be chemo-resistant [17]. A potent inhibition of cell growth of both cell lines was observed under the clavatustide B treatment. It is tentatively deduced that clavatustide B is a promising anti-cancer agent against chemo- and radio-therapy resistant cancers.

### 2.3. Evaluation of Cytotoxicity

**Figure 3:**
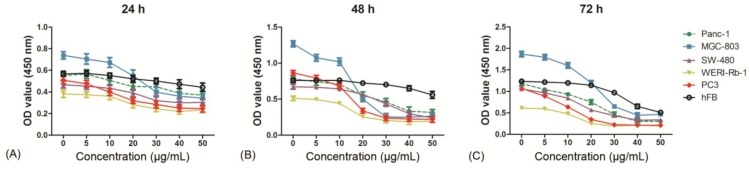
The evaluation of toxicity of clavatustide B by comparison of cell viability curves between normal human fibroblasts and cancer cell lines after 24 h (**A**); 48 h (**B**) and 72 h (**C**) treatments.

The toxicity of clavatustide B was evaluated *in vitro* with normal human fibroblasts (hFB). The results showed that treatment of hFB with 0–50 μg/mL of clavatustide B for 24 h and 48 h did not induce an obvious suppression of cell proliferation. In contrast, the cancer cell line showed a significant inhibition of cell proliferation by clavatustide B (Figure 3A,B). Viable cell number of hFB continued to increase during 48 h at 40–50 μg/mL of treatment by comparison of OD values, although a decrease in viable cell number was observed at 40–50 μg/mL compared with blank control at 72 h (Figure 3C). The results indicated that long-term treatment with clavatustide B (>72 h) with high concentration (>40 μg/mL) appeared to inhibit the growth of the normal human fibroblasts cells. However, since clavatustide B had hardly any effect on hFB cells even at Max effect dose for cancer cells with 48 h, it was inferred to be an anti-cancer agent with relatively low toxicity.

### 2.4. Clavatustide B Delays G1-S Cell Cycle Transition

**Figure 4:**
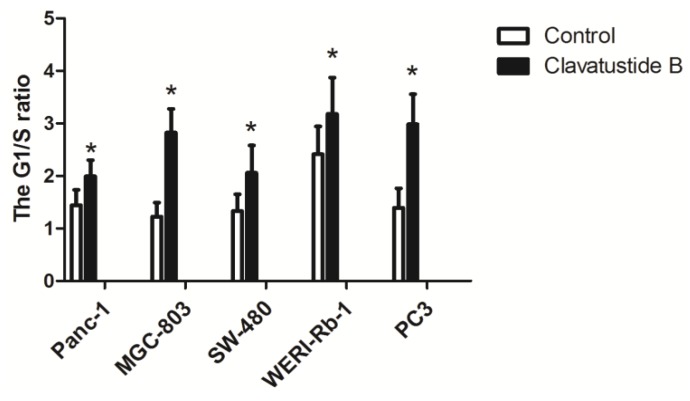
Clavatustide B increases G1/S phase ratio in Panc-1, MGC-803, SW-480, WERI-Rb-1 and PC3 cells. * *p* < 0.05.

Cell-cycle analysis was performed in HepG2 cells in our previous study, revealing an accumulation of HepG2 cells in G1 phase and reduction of cells in S phase induced by clavatustide B [2]. In the present study, the effect was confirmed in other cancer cell lines. The results showed that clavatustide B inhibited G1-S phase cell cycle transit in Panc-1, MGC-803, SW-480, WERI-Rb-1 and PC3 cells with the respective IC_50_ doses for 48 h (Figure 4).

The cell cycle can be divided into four phases, and the cellular decision to initiate mitosis or to become quiescent occurs during the G1 phase. Duration of G1 is highly variable and under the control of a complex network [18]. This phase is marked by occurrence of proteins required in S phase, when DNA replication happens. Genetic alterations in genes that regulate progression through G1 phase, make a major contribution to the development of human cancers [18]. In this study, clavatustide B was proved to delay G1-S phase cell cycle transition, exhibiting significant anti-cancer potency (Figure 4).

### 2.5. Clavatustide B Regulates G1-S Transition Genes

**Figure 5:**
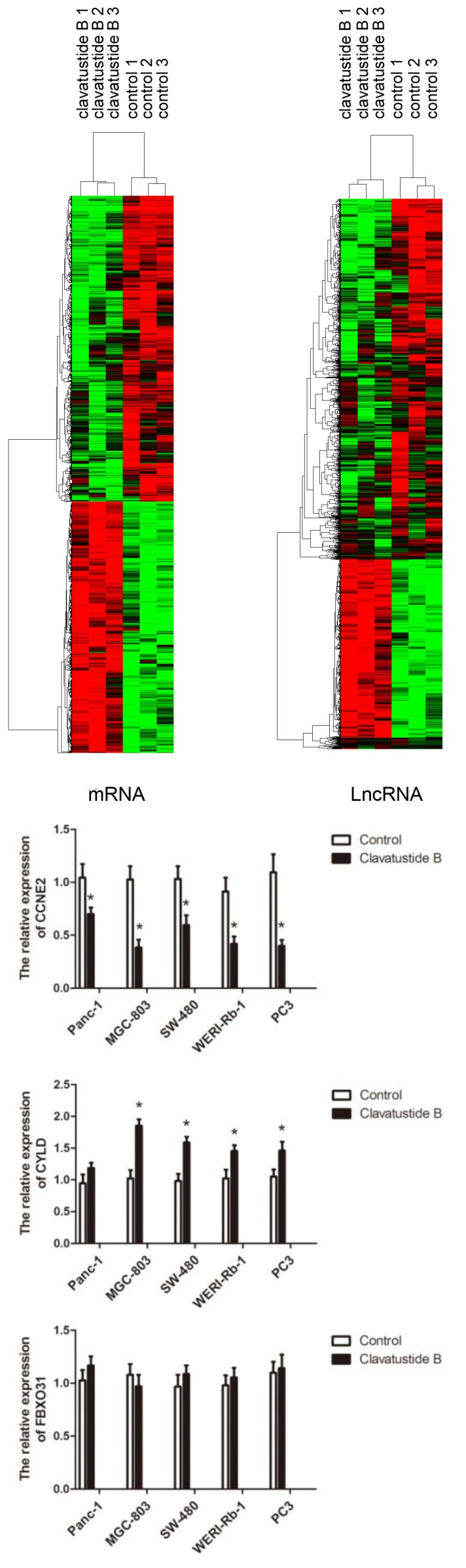
The differentially expressed genes between clavatustide B treated group and the control group. (**A**) The Heat Map showing differentially expressed mRNAs and long non-coding RNAs between clavatustide B treated group and control group in HepG2 cells; (**B**) Real time PCR comparing the expression of *CCNE2*, *FBXO31* and *CYLD* between clavatustide B treated group and control group in Panc-1, MGC-803, SW-480, WERI-Rb-1 and PC3 cells. * *p* < 0.05.

To further explore the mechanism of anti-proliferation effect, particularly the G1-S phase inhibition, Affymetrix GeneChip^®^ Human Genome U133 Plus 2.0 Array was used to screen transcriptome differences between clavatustide B treated and untreated HepG2 cells. Expression levels of 2622 genes and 3648 long non-coding RNAs in HepG2 cells were significantly altered after treatment at a concentration of 25 μg/mL clavatustide B for 48 h (Figure 5A). Since we were still at the beginning of the exploration of lncRNAs and their functional roles, we focused on the regulation of mRNAs in the presented study. Among the 2622 genes, 39 cell cycle-associated genes were significantly regulated. Genes such as *GSPT2*, *USP2*, *TXNIP*, *FBXO31*, *CYLD* and *CCNE2* are believed to involve in G1-S transition [18]. *CCNE2* was significantly down-regulated by 1.91-fold. In contrast, *FBXO31* and *CYLD* were significantly up-regulated by 1.45- and 1.43-fold, respectively. These genes were finally enrolled into the next real-time PCR verification for other cancer cell lines. The results showed that the expression of *CCNE2* was significantly lower in the clavatustide B treated group than that in the control group in all five cancer cell lines (Figure 5). The *CYLD* had higher expression in the clavatustide B treated group than that in the control group. However, it is not statistically significant in Panc-1 cells. No significant difference between the clavatustide B treated group and the control group in the expression of *FBXO31* was observed.

The G1-S cell cycle checkpoint plays a key role in the progression of cell division, controlling the passage of cells from G1 into the S phase. Dozens of genes involved in the G1-S checkpoint signaling pathway have been discovered, including *P16*, *P21*, *P53*, *MDM2*, *CCND1*, *CHEK2*, *CDC2*, *CDC6*, *CDK4* and *CDK6* [18,19,20]. *CCNE2* (cyclin E2) activates cyclin-dependent kinase 2 and controls progression through the G1-S checkpoint [21]. *CYLD*, a tumor suppresser gene, negatively regulates cell proliferation via a significant delay in the G1-S transition [22]. In the present study, down-regulation of *CCNE2* and up-regulation of *CYLD* by clavatustide B were observed, which would trap cancer cells at the G1-S checkpoint and prevent them from initiating DNA replication. These findings provided molecular evidence that clavatustide B reduced cancer cells growth and division, which might account for its anti-cancer potential.

## 3. Experimental Section

### 3.1. General Experimental Procedures

Optical rotations were recorded on a Perkin-Elmer-341 polarimeter. The IR spectra (CHCl_3_) were run on a NicoletAvatar-360FT-IR spectrometer. ^1^H NMR (500 MHz) and ^13^C NMR (125 MHz) spectra were measured at 25 °C on a Bruker AVANCE DMX 500 NMR spectrometer with TMS as internal standard. TOF-MS were recorded on a AB Sciex Triple TOF-MS spectrometer. ESIMS were recorded on an Agilent 6460 Triple Quad LC/MS. TLC was performed using Merck precoated plates (Silica gel 60 F254) of 0.25 mm thickness. Sephadex LH-20 (Amersham, Stockholm, Sweden) was used for column chromatography.

### 3.2. Fungal Cultivation and Stress Applications

The fungus *A. clavatus* C2WU was separated from the marine *X. testudinatus*, which was collected from Taiwan Kueishantao hydrothermal vent, and identified by its ITS-5.8s rDNA sequences. Subcultures of the organism are deposited at the Ocean College, Zhejiang University. Cultures were separated into control (5 L) and stressed groups (5 L). The control fungus was grown in the potato culture medium in sterilized and filtrated natural seawater. The stressed culture medium consisted of an additional 50 μmol/L ZnSO_4_. The fermentations were carried out at 24 °C for 10 days.

### 3.3. Extraction and Isolation

The whole culture of the control and stressed broth of *A. clavatus* C2WU (5 L) were filtered. The air-dried mycelia of control (24 g) and the stressed group (20 g) were extracted at room temperature with MeOH (3 × 1 L), respectively. The extracts were evaporated *in vacuo* to afford a gummy residue for both Zn treated (1.8 g) and corresponding controls (2.0 g). The residues were partitioned in H_2_O (500 mL) and extracted successively with EtOAc (3 × 500 mL) and *n*-butanol (3 × 500 mL). The EtOAc and *n*-butanol extracts of the treated and control cultures were subjected to TLC examination on aluminium sheets pre-coated with Silica gel 60 F254 (Merck, Darmstadt, Germany). The spots were applied in as equal amounts as possible. The plates were developed in the following developing solvent systems: benzene–acetone (7:1), benzene–EtOAc (5:1), petroleum ether–EtOAc (5:1) for the EtOAc extract; CHCl_3_–MeOH (3:1), CH_2_Cl_2_–MeOH (4:1) and benzene–CHCl_3_–MeOH (1:3:1) for the *n*-butanol extract. After development, the plates were examined under UV light (250 nm) to locate any additional spots in the different extracts of the treatments in comparison with those of the corresponding control extracts. The spots on the plates were also visualized by spraying with an EtOH-H_2_SO_4_ solution. One additional compound was detected on the plates developed in benzene–acetone (7:1) solvent system in the EtOAc extract of stress elicited mycelium. Several prep-TLC plates were prepared and the target compound was isolated by preparative TLC in the developing solvent systems of benzene–acetone (7:1). The crude compounds were applied to a Sephadex LH-20 column (1 × 80 cm, 38 g, Amersham), and eluted with acetone to yield pure compound **1** (5.3 mg). Compound **2** was prepared by the methods reported [2].

### 3.4. Cell Culture and Proliferation Assay

Human liver cancer (HepG2), pancreatic cancer (Panc-1), gastric cancer (MGC-803), colorectal cancer (SW-480), retinoblastoma (WERI-Rb-1), prostate cancer (PC3) and human fibroblasts (hFB) were purchased from the Cell Bank of the Chinese Academy of Sciences (Shanghai, China), and cultured in DMEM or RPMI-1640 with 10% fetal bovine serum at 37 °C in a humidified atmosphere containing 5% CO_2_. Viable cell number was determined by cell counting kit-8 (Dojindo, Tokyo, Japan). Briefly, cells were plated in 96-well plate (2 × 10^3^ cells per well) and incubated for 24 h. Then the cells were treated with clavatustide B for different time points and concentrations. After incubation, CCK-8 solution was added and the absorbance at 450 nm was measured according to the manufacturer’s instructions. The experiment was independently repeated three times.

### 3.5. Cell Cycle Assay

Cell cycle analysis was performed using flow cytometry as we described before [23]. Briefly, cells were washed and fixed in 70% cold ethanol overnight at 4 °C. Then cell was mixed with 0.5 mL DNA Prep LPR (Beckman Coulter, Fullerton, CA, USA) in the dark for 20 min. Cell cycle analysis was performed on the same flow cytometer (Cytomics FC 500, Beckman Coulter, Miami, USA). ModFit LT software (Verity Software House, Maine, USA) was used to calculate the percentage of cell population in S, G1 and G2 phase.

### 3.6. Microarray Assay

Cellular RNA from HepG2 cells was extracted using Trizol (Invitrogen, Carlsbad, CA, USA), and quality controlled as directed with the Affymetrix expression technical manual. RNA (25 ng) was used to produce biotin-labeled cRNA, which was hybridized to Affymetrix GeneChip^® ^Human Transcriptome Array 2.0. Array washing, scanning and probe quantification protocols were carried out according to the manufacturer’s instructions using Affymetrix GeneChip Operating Software (GCOS). For each array, GCOS output was imported as CEL files into Partek Genomic Suite software (Agilent, Palo Alto, CA, USA), and gene expression data quantified with the RMA (Robust Multichip Averaging) algorithm, normalized and corrected for multiple testing with random variance model (RVM) and Benjamin and Hochberg method for detection of differentially expressed genes and false discovery rate, respectively [23]. Fold change = Geom mean of intensities in clavatustide B group/Geom mean of intensities in control group.

### 3.7. Real-Time PCR

Cellular RNA was extracted from Panc-1, MGC-803, SW-480, WERI-Rb-1 and PC3 cells using Trizol (Invitrogen, Carlsbad, CA, USA) according to the manufacturer’s instructions. Total RNA was reverse-transcribed using Superscript III reverse transcriptase and oligo (dT) primer. Real time PCR was performed and analyzed in ABI PRISM 7500 Sequence Detection System (Applied Biosystem, Carlsbad, CA, USA) with SYBR green ready mix (Applied Biosystem) and SDS 2.1 software (Applied Biosystems) as described before [24]. All reactions were measured in triplicates in a final volume of 10 μL. Cycling conditions were chosen according to the manufacturer’s protocols. The relative level of mRNA was calculated against GAPDH (internal control) using the 2^−ΔΔCt^ method in each cell line, respectively.

### 3.8. Statistical Analysis

SPSS for Windows version 13.0 (SPSS Inc., Chicago, IL, USA) was used for statistical analysis. *P* values of less than 0.05 were considered statistically significant. Values in the present study were reported as Mean ± SD from three independent experiments repeats. Two-sided student’s unpaired test was used for comparison.

## 4. Conclusions

Marine hydrothermal vent microbial habitats are strongly influenced by elevated levels of metals. Hydrothermal microorganisms respond rapidly to changes in the concentrations and availability of metals within their environment, where geothermally heated water reacts with its host rock forming fluids that nourish a diverse array of geothermally dependent microorganisms [1]. In this study, small molecule clavatustide C was successfully induced by Zn. Clavatustide B, the product secreted both in the normal and metal spiked cultures of *A. clavatus* C2WU, exhibited a potent anti-cancer effect in various human cancers, including liver cancer, pancreatic cancer, gastric cancer, colorectal cancer, prostate cancer and retinoblastoma. Clavatustide B inhibited the proliferation of cancer cells via a remarkable delaying of G1-S cell cycle transition. The G1-S cell cycle checkpoint-associated molecules including Cyclin E2 played key roles in the clavatustide B-induced cell cycle blocking. The results showed that clavatustide B demonstrated a potent anti-proliferation effect in the chemo-resistant cell lines, such as Panc-1. Thus, clavatustide B was proved to be a promising anticancer candidate, particularly for the treatment of chemoradiotherapy resistant cancers.
